# The correlation between triglyceride-glucose index in early pregnancy (<20 weeks) and pregnancy complications and adverse pregnancy outcomes: a systematic review and meta-analysis

**DOI:** 10.3389/fmed.2026.1811358

**Published:** 2026-04-23

**Authors:** Wenli Zhang, Junzhi Wang, Liangliang Chu, Xinyu Pi, Guochun Zhang

**Affiliations:** 1 Shandong First Medical University (Shandong Academy of Medical Sciences), School of Nursing, Jinan, China; 2 Shandong Provincial Qianfoshan Hospital Department of Obstetrics, Jinan, China

**Keywords:** adverse pregnancy outcomes, early pregnancy, meta-analysis, pregnancy complications, systematic review, triglyceride-glucose index

## Abstract

**Background:**

Insulin resistance in the early pregnancy stage can independently lead to the occurrence of gestational diabetes mellitus and adverse pregnancy outcomes. To evaluate the relationship between the Triglyceride-Glucose (TyG) index in early pregnancy and pregnancy complications and adverse pregnancy outcomes using meta-analysis.

**Methods:**

Search the CNKI, Wanfang, VIP, China Biomedical Literature database, PubMed, Embase, Web of Science, and the Cochrane Library. The search covered the period from the establishment of each database to December 28, 2025. Two researchers using the Newcastle-Ottawa Scale to assess the quality of the included studies and extracted data. Statistical analysis was performed using RevMan 5.4 and Stata 17.0.

**Results:**

Twenty-three studies were included, involving 220,985 participants with 61,774 exposed individuals. Meta-analysis revealed that compared with low TyG index in early pregnancy, high TyG index was significantly associated with increased risks of gestational diabetes mellitus, gestational hypertension, preeclampsia, preterm birth, large for gestational age, and macrosomia (*p* < 0.05).

**Conclusion:**

A high TyG index in early pregnancy is significantly positively associated with the occurrence of gestational diabetes, gestational hypertension, preeclampsia, preterm birth, large for gestational age, and macrosomia. The TyG index can serve as a simple and reliable indicator for the early diagnosis of high-risk pregnancies in pregnant women, facilitating timely clinical intervention by healthcare providers to reduce the incidence of pregnancy complications and adverse pregnancy outcomes.

**Systematic review registration:**

https://www.crd.york.ac.uk/PROSPERO/view/CRD420261293271, Identifier CRD420261293271.

## 1 Introduction

In recent years, with the increasing proportion of older pregnant women, obese/overweight pregnant women, and multiparous women, the severity of maternal metabolic disorders has intensified. Excessively released inflammatory factors can cause maternal genetic damage, imbalance in antioxidant levels, and impaired insulin sensitivity, leading to pathological insulin resistance (IR) that severely impacts maternal and fetal health (1). Research indicates that early pregnancy IR independently influences the occurrence of gestational diabetes mellitus (GDM), hypertensive disorders of pregnancy (HDP), and adverse pregnancy outcomes (APOs) (2, 3). The global incidence of GDM currently reaches 15%, while approximately 5%–10% of pregnant women suffer from HDP (4). These pregnancy complications not only significantly increase the risk of APOs such as placental abruption, low birth weight (LBW), and fetal distress, but also constitute risk factors for mothers developing chronic diabetes, chronic hypertension, and cardiovascular diseases in the future (5). Research has indicated that diagnosing and treating women with GDM before 20 weeks of gestation significantly reduces the incidence of adverse neonatal outcomes, such as birth trauma, respiratory distress, phototherapy, stillbirth, neonatal death, and shoulder dystocia, compared to the absence of timely intervention (6). Currently, screening and diagnosis for pregnancy complications frequently occur after 20 weeks, by which time the foetus has already been subjected to an adverse intrauterine environment for a prolonged duration, treatment cannot fully mitigate the detrimental effects on the fetus. Therefore, implementing early pregnancy screening (<20 weeks) and developing effective prevention strategies, along with identifying optimal indicators for predicting pregnancy complications and APOs, are particularly crucial. The TyG index, based on fasting blood glucose and triglycerides, is widely recognized as a simple, cost-effective, and reliable biomarker for assessing IR and the risk or prognosis of metabolic disorders. In recent years, the association between the TyG index in early pregnancy and pregnancy complications such as GDM and HDP has drawn significant attention (7, 8). However, evidence linking TyG to other potential APOs—including premature birth (PTB), large/small for gestational age (LGA/SGA), macrosomia, LBW, fetal distress, preterm premature rupture of membranes (PPROM), cesarean section and placental abruption—remains limited and often inconsistent. Therefore, this study conducted a meta-analysis to integrate the relationship between the TyG index in early pregnancy and the occurrence of pregnancy complications and APOs, aiming to provide valuable reference for improving maternal and infant outcomes.

## 2 Materials and methods

### 2.1 Literature

PubMed, Web of Science, Embase, the Cochrane Library, CNKI, Wanfang, VIP and China Biomedical Literature database were searched from their inception to December 2025. The following search terms are used:"TyG index” OR “triglyceride glucose index” OR “triacylglycerol glucose index” OR “Pregnancy Trimester, First” OR “Pregnancy complications”.

### 2.2 Inclusion and exclusion criteria inclusion criteria

1) Study Design: Prospective or retrospective observational studies; 2) Study Population: Single-pregnancy cases with age≥18 years; 3) Study measure: TyG index calculated from fasting plasma triglycerides (TG) and fasting blood glucose (FBG) using the formula In[TG(mg/dL) × FBG(mg/dL)/2] (9). Individuals with high TyG index were defined as the experimental group, while those with low TyG index formed the control group. TyG index data were collected during weeks 0–20 of pregnancy. 4) Outcome measures: The primary outcomes were pregnancy complications, including GDM, preeclampsia (PE), and gestational hypertension (GH). The secondary outcomes were the incidence of APOs, including PTB, LGA/SGA, PPROM, LBW, fetal distress, macrosomia, caesarean section, and placental abruption. Exclusion criteria: 1) Article type: Conference abstracts, newspapers, laboratory studies, review articles, etc. 2) Duplicate publications; non-Chinese or non-English literature. 3) Literature with missing data, unobtainable data, or inaccessible full text. 4) Individuals with pre-existing hypertension, diabetes, other adverse metabolic disorders, or concomitant severe systemic diseases such as cardiovascular, respiratory, digestive, urinary, or autoimmune diseases.

### 2.3 Literature screening and data extraction

Endnote software was used to identify duplicate publications. Secondary screening and manual deduplication were conducted based on predefined inclusion and exclusion criteria. An Excel spreadsheet was created to extract information from eligible studies. Extracted content primarily included: 1) Basic study information: first author, publication year, country, sample size, study type, TyG analysis type, high TyG index range, diagnostic criteria for pregnancy complications, adjusted confounding factors, TyG measurement timing, and outcome measures; 2) Subject characteristics: age, etc.; 3) Factors related to bias risk assessment, including NOS scores.

### 2.4 Quality assessment

The Newcastle-Ottawa Scale (NOS) was employed to independently evaluate the quality of the included studies. Two researchers conducted the assessments independently, and any disagreements were resolved through discussion with a third researcher. The assessment criteria included study population selection, comparability of groups, and the exposure-outcome relationship. The scale has a total score of 9 points. This study only included medium-to-high quality literature, defined as studies with a score of ≥6 points (10).

### 2.5 Statistical analysis

Statistical analysis was performed using RevMan 5.4. The odds ratio (OR) was used as the effect measure, with point estimates and 95% confidence intervals (CI) provided for each effect size. In original studies with multiple models in multivariate analysis, the model best adjusting for confounders was selected. The TyG index could be extracted and synthesized as either a continuous or categorical variable. When the original study assessed the TyG index as a categorical variable, the estimated value of the highest quartile relative to the first quartile was extracted. When the TyG index was assessed as a continuous variable, the estimated value per unit increase of the variable was extracted. Prior to data pooling, log-transform the OR values and 95% CI to ensure normality. In this study, the selection of the effect model was primarily informed by clinical and methodological considerations. A fixed-effect model was employed when it was assumed that the relative treatment effects across the included studies were consistent. Conversely, a random-effects model was utilized when it was anticipated that the true treatment effects among the included studies were heterogeneous. Furthermore, the *I^2^* statistic was employed to quantify the degree of statistical heterogeneity among the studies. For outcome measures exhibiting significant heterogeneity (*I^2^*>50%), additional methods, such as subgroup or sensitivity analyses, were implemented to investigate and address potential sources of heterogeneity (11).

## 3 Results

### 3.1 Literature search results

**Figure 1:**
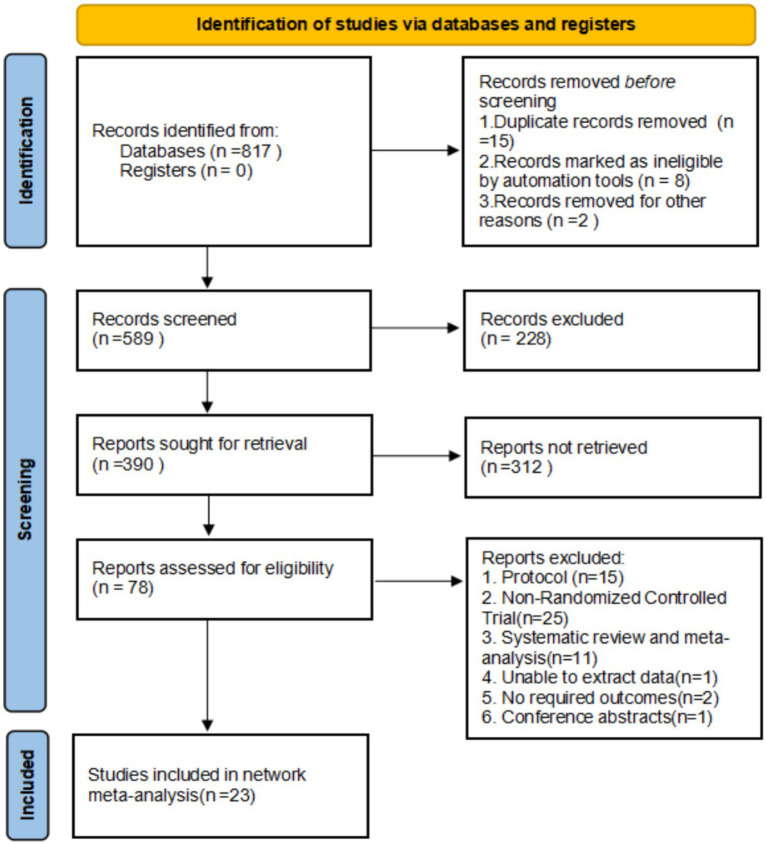
Literature screening. Source: Page et al. (60). This work is licensed under CC BY 4.0. To view a copy of this license, visit https://creativecommons.org/licenses/by/4.0/.

Following the inclusion and exclusion criteria, 23 studies were ultimately included. See Figure 1.

### 3.2 Basic characteristics and quality assessment results of included studies

A total of 23 studies were included (12–34), originating from countries including India, China, Iran, Mexico, and South Korea. The study population comprised 220,985 subjects, with 61,774 exposed individuals. Except for Tankasali (12), all included studies adjusted for confounding factors and achieved NOS scores of 7 or 8. Among these, 13 studies (12, 14, 15, 19, 21, 22, 24–27, 32–34) scored 7 points, and 10 studies (13, 16–18, 20, 23, 28–31) scored 8 points, indicating good quality of the literature, as shown in Table 1.

**Table 1:** Basic characteristics of included literature.

Research type	Study type	Type of analysis	Experimental group/control group	High TyG index range	Age	Country	Adjusted factors	Diagnostic criteria for pregnancy complications	Outcome measures	Collection time	Nos
Tankasali et al., 2025 (12)	Prospective	Categorical variables	34/236	—	25.9 ± 4.6	India	—	DIPSI	GDM	<20	7
Song et al., 2025 (13)	Retrospective	Categorical variables	1842/10100	≥8.62	30.1 ± 4.1	China	Age, education level, pre-pregnancy BMI, parity, gestational age, SBP, DBP, TC, LDL, HDL, HbA1c, TP, ALB	IADPSG	GDM	≤14	8
			94/11848					ACOG	PE		
			418/11524					ACOG	GH		
			306/11636					—	PTB		
			378/11564					—	Macrosomia		
			163/11779					—	LBW		
Pazhohan et al., 2017 (14)	Prospective	Categorical variables	176/778	≥8.99	24.4 ± 3.2	Iran	Age, family history of diabetes, early pregnancy BMI, history of GDM	IADPSG	GDM	<12	7
			248/706					—	LGA		
Gurza et al., 2025 (15)	Prospective	Categorical variable	55/278	≥8.60	30.8 ± 5.1/28.9 ± 5.4	Mexico	Age, pre-pregnancy weight, GWG, BMI	IADPSG	GDM	≤14	7
			34/209					ACOG	PE		
			29/304					—	LGA		
			20/313					—	SGA		
			41/292					—	PTB		
			11/322					ACOG	GH		
			88/150					—	Cesarean section		
Lin et al., 2023 (16)	Prospective	Categorical variables	1381/10727	≥8.68	30.2 ± 3.9	China	Age, education level, parity, gestational age at delivery, pre-pregnancy BMI, infant Sex, smoking status, DBP, HDL-C, LDL-C, TC	—	LGA	<13	8
He et al., 2025 (17)	Retrospective	Categorical variables	409/2438	>8.43	31.8 ± 3.7	China	Age, BMI, TC, LDL-C, UA, HDL-C, fasting Glucose, UA, Cr, SAT, AST	IADPSG	GDM	6–10	8
			90/2757					ACOG	GH		
			81/2766					ACOG	PE		
			155/2692					—	PTB		
			157/2690					—	Macrosomia		
			26/2821					—	LBW		
			674/2173					—	PPROM		
			1144/1703					—	Cesarean section		
Sánchez-García et al., 2020 (18)	Prospective	Categorical variable	29/135	> 8.70	24.9 ± 5.4/25 ± 5.2	Mexico	Early pregnancy BMI, parity, family history of diabetes, DBP	IADPSG	GDM	≤14	8
Cui et al., 2025 (19)	Retrospective	Categorical variables	1812/6793	> 8.63	30.0 ± 4.1	China	Age, BMI, education level, family history of diabetes, parity	IADPSG	GDM	≤14	7
			376/8229					—	PTB		
			377/8228					—	LBW		
			304/8301					ACOG	HDP		
			142/8463					ACOG	PE		
			977/7628					—	LGA		
			458/8147					—	SGA		
			252/8353					—	Macrosomia		
Li et al., 2022 (20)	Prospective	Categorical variables	2787/8600	>8.88	30.0 ± 4.0	China	Race, pre-pregnancy BMI, age, assisted reproductive technology, history of miscarriage, parity, gravidity, gestational age at delivery, GWG, gestational age, SBP, BDP, TC, LDL-C, HDL-C, HbA1c, creatinine, ALT, AST	IADPSG	GDM	≤14	8
			387/11000					ICD-10 O14	HDP		
			196/11191					ICD-10 O13	PE		
			257/11130					—	Placental abruption		
			2152/9235					—	Fetal distress		
			3014/8373					—	PPROM		

Research	Study type	TyG analysis type	Experimental group/control group	High TyG index range	Age	Country	Adjusted factors	Diagnostic criteria for pregnancy complications	Outcome measures	Collection time	Nos
Pan et al., 2023 (21)	Retrospective	Categorical variables	289/861	>8.70	29.7 ± 5.3/28.2 ± 5.6	China	Age, pre-pregnancy BMI, family history of hypertension, parity	ACOG	HDP	9–12	7
			49/1101					—	LBW		
			57/1093					—	Fetal distress		
Li et al., 2024 (22)	Retrospective	Categorical variables	10,680/57256	≥8.65	31.0 ± 3.9	China	Age, education level, parity, health insurance status	IADPSG	GDM	9–14	7
			4104/63832					ACOG	HDP		
			6021/61915					—	LGA		
			3449/64487					—	PTB		
Liu et al., 2020 (23)	Prospective	Categorical variable	66/286	≥8.30	30.4 ± 4.0/29.0 ± 3.5	China	Age, education level, physical activity level, pre-pregnancy BMI, parity, family history of diabetes, history of polycystic ovary syndrome, CRP, fetal sex, gestational age, GWG	IADPSG	GDM	<12	8
			31/321					—	LGA		
Guo et al., 2024 (24)	Prospective	Categorical variables	447/1195	≥9.29	31.7 ± 4.0	China	Race, pre-pregnancy BMI, Age, assisted reproductive technology, history of miscarriage, gestational order, parity, gestational age at delivery, GWG, SBP, DBP, TC, LDL-C, HDL-C, HbA1c, UA, Car, ALT, AST	IADPSG	GDM	≤14	7
			88/1536					ISSHP	GH		
			41/1583					ISSHP	PE		
			34/1590					-	Placental abruption		
			209/1415					-	Fetal distress		
			60/1564					-	PPROM		
Du et al., 2024 (25)	Prospective	Categorical variable	329/1348	>8.32	31.5 ± 3.2	China	Age, pre-pregnancy BMI, family history of diabetes, adverse pregnancy history, early pregnancy laboratory markers, FINS, TC, HDL-C, LDL-C	China HIP 2022	GDM	6–12	7
Liu et al., 2025 (26)	Prospective	Categorical variable	128/290	>8.65	31.3 ± 4.4/30.3 ± 3.7	China	Age, pre-pregnancy BMI, SBP, DBP, family history of diabetes, smoking history, alcohol consumption history, parity status	China HIP 2022	GDM	≤14	7
Zhang et al., 2025 (27)	Retrospective	Categorical variables	102/529	—	28.1 ± 3.0	China	Age, BMI, SBP, DBP, parity, history of miscarriage, TA, HDL, LDL, TBIL, UA	IADPSG	GDM	5–8	7
Mo et al., 2024 (28)	Prospective	Categorical variables	36/553	>8.51	32.1 ± 3.8	South Korea	Age, pre-pregnancy BMI, parity, hepatic steatosis, AST, GGT, ALT, TC, HDL-C, LDL-C, and insulin	Carpenter and coustan	GDM	10–14	8
Zhang et al., 2025 (29)	Prospective	Categorical variable	6045/40947	≥ 8.45	34.5 ± 3.9	China	Age, parity, pre-pregnancy BMI, family history of diabetes, history of GDM, education level, CHO and LDL-C	IADPSG	GDM	6–14	8
Zhao et al., 2024 (30)	Prospective	Categorical variables	121/1211	≥ 6.73	31.6 ± 3.9/30.9 ± 3.8	China	Age, BMI, parity, SBP, ALT, UA, CRP	—	Macrosomia	<8	8
Li et al., 2025 (31)	Retrospective	Categorical variables	2308/37078	>9.75	32.2 ± 4.4	China	Age, BMI, SBP, DBP, aspirin use, antihypertensive use, smoking, alcohol consumption, IVF, ALT, UA, Cr, TC, Education Level	ACOG	PE	<20	8
			2306/37080					—	PTB		
			2208/37178					—	LBW		
Gao C et al., 2023(32)	Retrospective	Continuous variable	1303/6904	—	33.6 ± 4.0/31.7 ± 3.7	China	Age, pre-pregnancy BMI, TC	China HIP 2022	GDM	6–14	7
Han et al., 2025 (33)	Retrospective	Continuous variables	41/1003	—	30 ± 2.5/32 ± 3.5	China	Age, pre-pregnancy BMI, TG, HDL-C, LDL-C	—	PE	8–10	7
Jin et al., 2025 (34)	Retrospective	Continuous variables	189/830	—	—	China	Age, parity, pre-pregnancy BMI, total cholesterol (TC), low-density lipoprotein cholesterol (LDL-C)	China HIP 2022	GDM	6–14	7

SBP, Systolic Blood Pressure; DBP, Diastolic Blood Pressure; TC, Total Cholesterol; LDL, Low-Density Lipoprotein; HDL, High-Density Lipoprotein; HDL-C, High-Density Lipoprotein Cholesterol; LDL-C, Low-Density Lipoprotein Cholesterol; HbA1c, Glycated Hemoglobin; TP, Total Protein; ALB, Albumin; GWG, Gestational Weight Gain; UA, Uric acid; CAR, C-reactive protein to albumin ratio; ALT, Alanine aminotransferase; AST, Aspartate aminotransferase; GGT, Gamma-glutamyl transferase; TG, Fasting plasma triglycerides Triglycerides; TA, Total albumin; Cr, Creatinine; CHO, Cholesterol; IVF, In vitro fertilization; CRP, C-reactive protein; TP, Total Protein; FINS, Fasting Insulin; SAT, Subcutaneous Adipose Tissue; TBIL, Total Bilirubin; IADPSG, International Association for the Study of Diabetes in Pregnancy; ACOG, American College of Obstetricians and Gynecologists; ISSHP, International Society for the Study of Hypertensive Disorders in Pregnancy; DIPSI, Indian Diabetes in Pregnancy Study Group; ICD-10, International Classification of Diseases, 10th Revision China HIP 2022; Guidelines for Diagnosis and Management of Gestational Hyperglycemia 2022.

### 3.3 Meta-analysis results

#### 3.3.1 Correlation between early pregnancy TyG index and pregnancy complications

**Figure 2:**
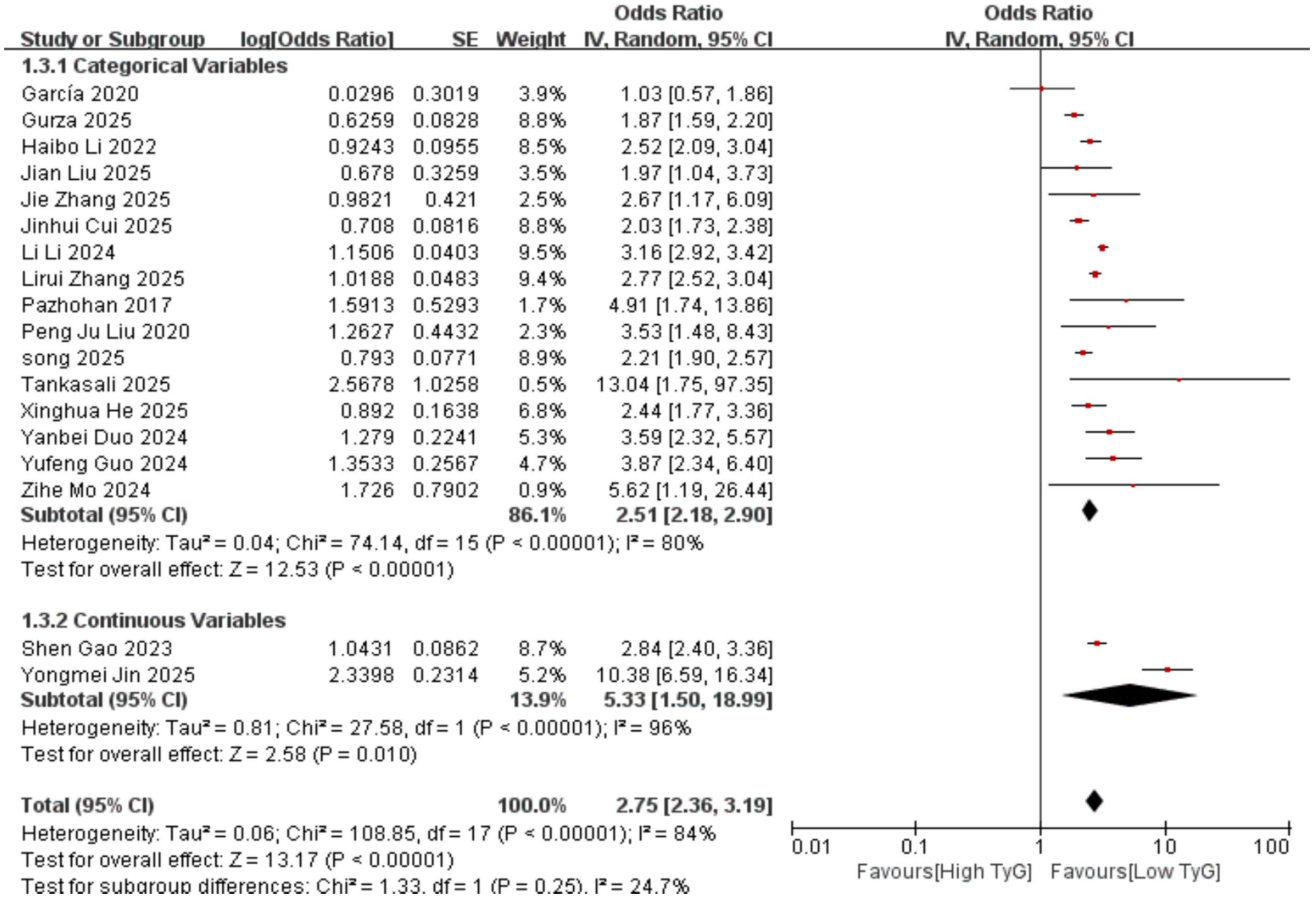
Forest plot of the relationship between TyG index in early pregnancy and the occurrence of GDM.

**Figure 3:**
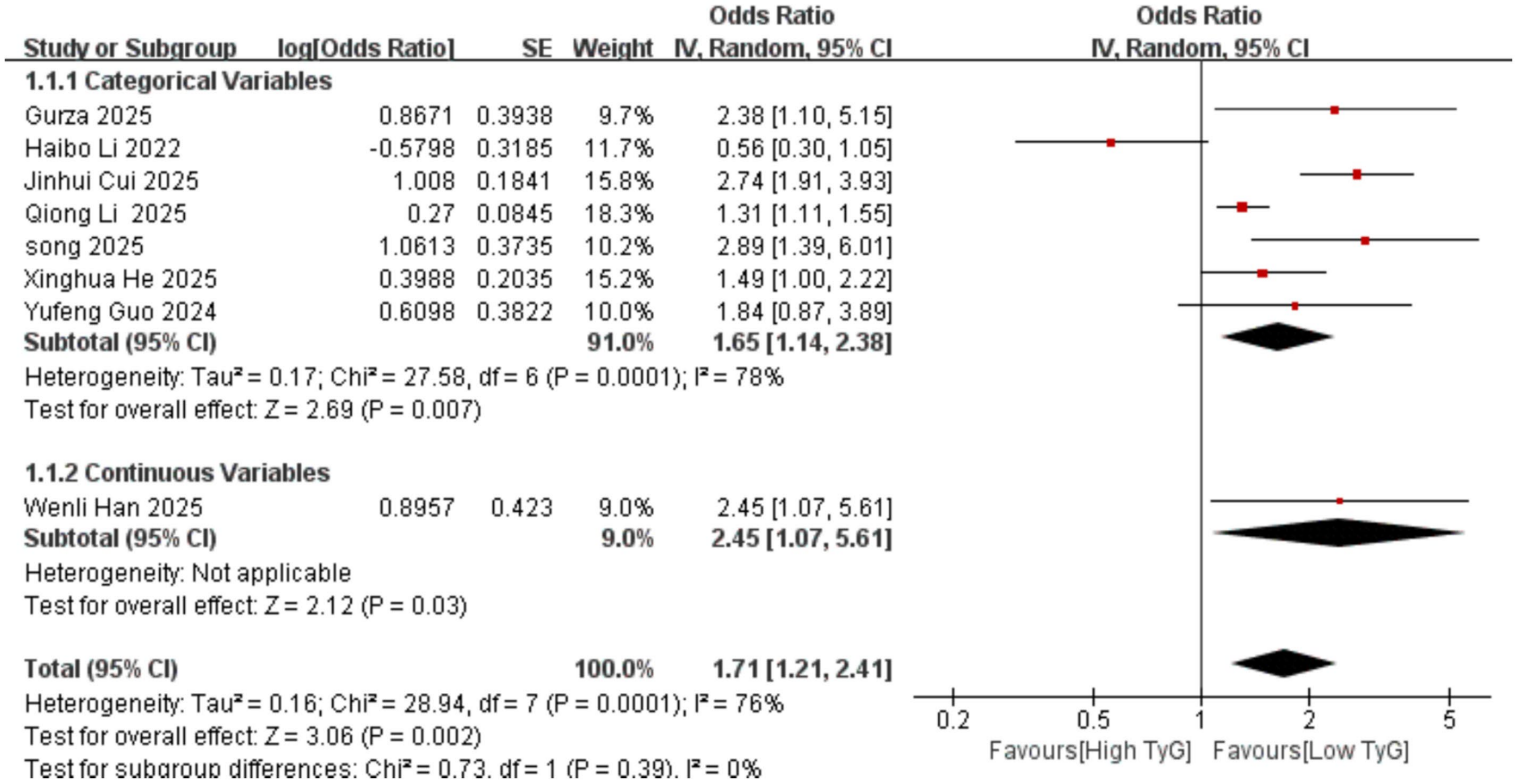
Forest plot of the relationship between the TyG index in early pregnancy and the occurrence of PE.

**Figure 4:**
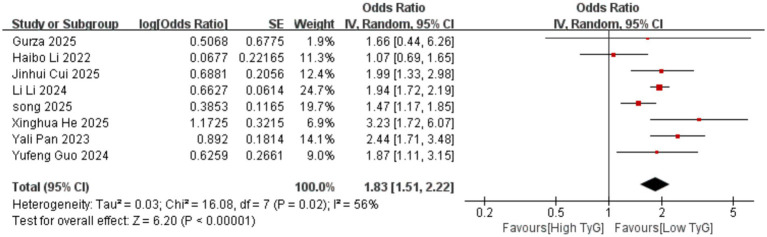
Forest plot showing the relationship between TyG index in early pregnancy and GH occurrence.

1. Correlation between early pregnancy TyG Index and GDM: Two studies evaluated the correlation between the TyG index as a continuous variable and GDM occurrence. Significant heterogeneity existed among the included results (*I^2^* = 96%). Therefore, a random-effects model was employed. Results indicated that compared with women with low TyG indices in early pregnancy, those with high TyG indices had a significantly higher risk of developing GDM [OR = 5.33, 95% CI (1.50, 18.99), *p* = 0.01]. Sixteen studies evaluated the association between the TyG index as a categorical variable and GDM occurrence. Significant heterogeneity existed among the included results (*I^2^* = 80%), necessitating the use of a random-effects model for analysis. Results demonstrated a significant positive correlation between early pregnancy TyG and GDM [OR = 2.51, 95% CI (2.18, 2.90), *p* < 0.00001] See Figure 2.
2. Correlation between TyG index in early pregnancy and PE: One study evaluated the correlation between the TyG index as a continuous variable and the occurrence of PE. The results showed that compared with women with low TyG index in early pregnancy, those with high TyG index were more likely to develop PE, with a statistically significant difference [OR = 2.45, 95% CI (1.07, 5.61), *p* = 0.03]. Seven studies evaluated the correlation between the TyG index as a categorical variable and the occurrence of PE. Significant heterogeneity existed among the included results (*I^2^* = 78%), thus a random-effects model was employed for analysis. Results indicated a significant positive correlation between the TyG index in early pregnancy and PE [OR = 1.65, 95% CI (1.14, 2.38), *p* = 0.007] See Figure 3.
3. Correlation between the TyG index in early pregnancy and GH: Eight studies evaluated the association between the TyG index as a categorical variable and the occurrence of GH. Heterogeneity existed among the included results (*I^2^* = 56%), thus a random-effects model was applied. Results indicated that compared with women with low TyG index in early pregnancy, those with high TyG index were more likely to develop GH, with a statistically significant difference [OR = 1.83, 95% CI (1.51, 2.22), *p* < 0.00001] See Figure 4.

#### 3.3.2 Correlation between early pregnancy TyG index and APOs

**Figure 5:**
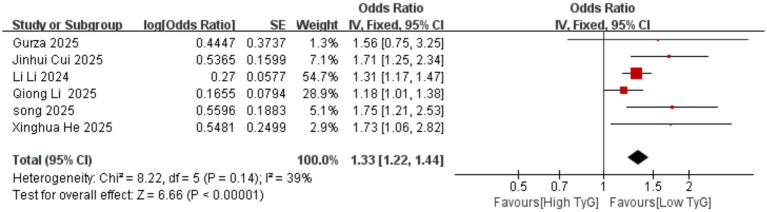
Forest plot showing the relationship between TyG index in early pregnancy and the occurrence of PTB.

**Figure 6:**
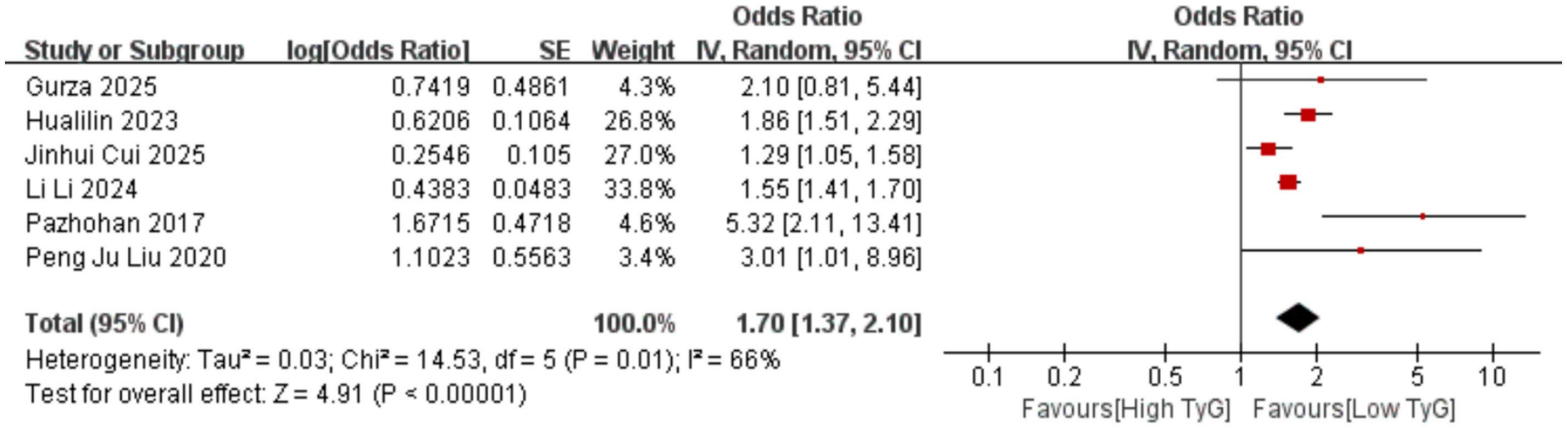
Forest plot showing the relationship between early pregnancy TyG index and LGA occurrence.

**Figure 7:**
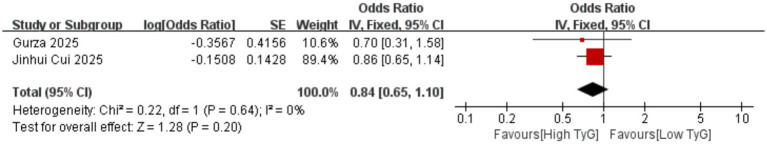
Forest plot showing the relationship between TyG index in early pregnancy and the occurrence of SGA.

**Figure 8:**
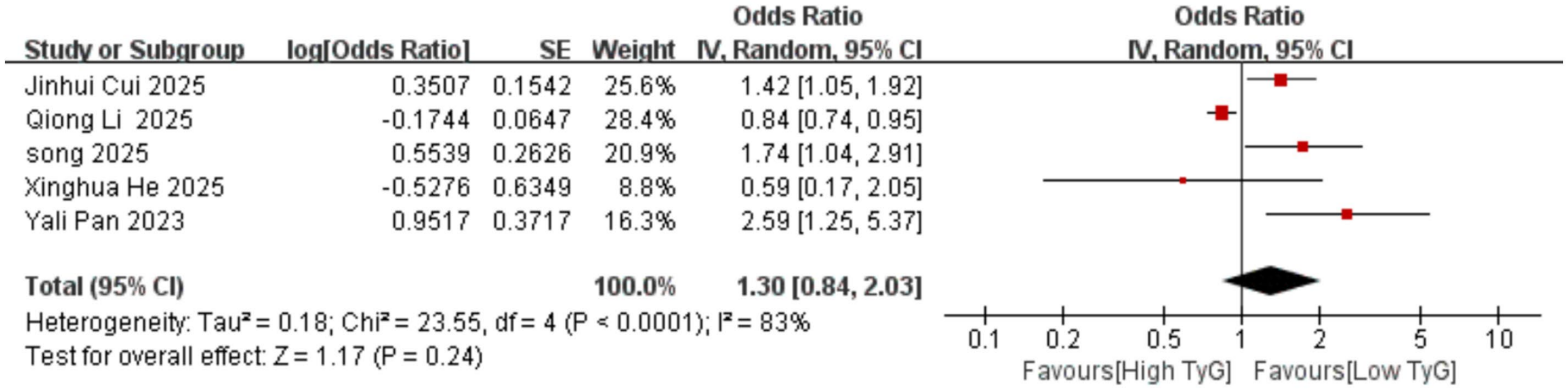
Forest plot showing the relationship between TyG index in early pregnancy and the occurrence of LBW.

**Figure 9:**
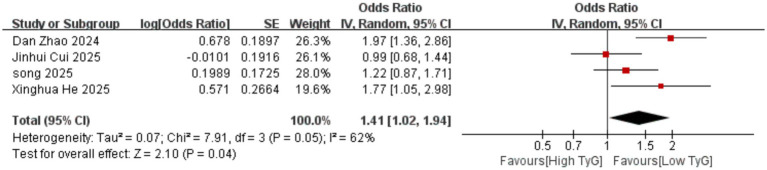
Forest plot showing the relationship between TyG index in early pregnancy and the occurrence of macrosomia.

**Figure 10:**
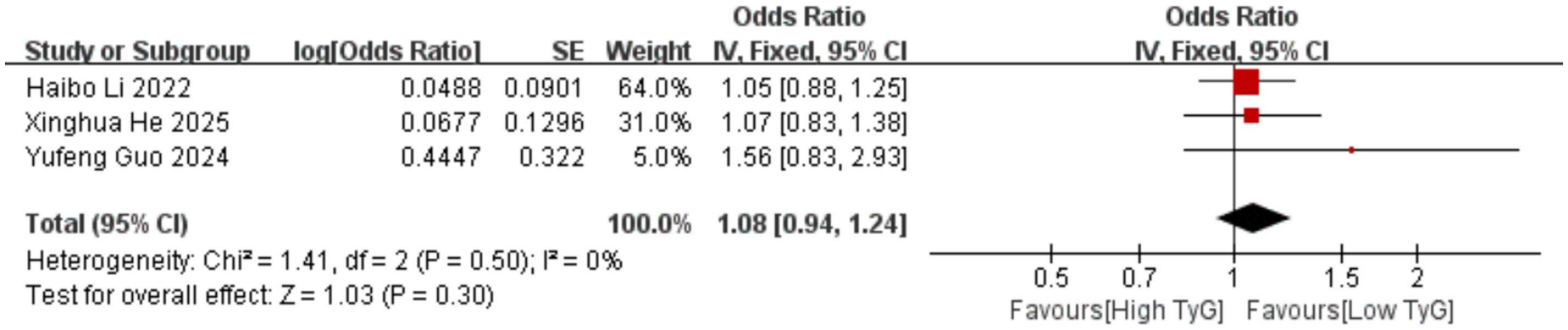
Forest plot showing the relationship between TyG index in early pregnancy and the occurrence of PPROM.

**Figure 11:**
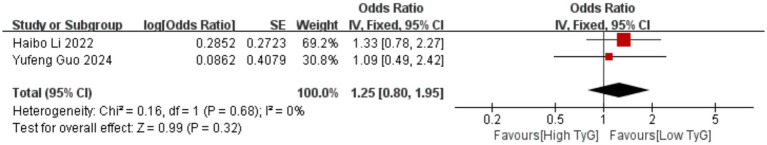
Forest plot showing the relationship between TyG index in early pregnancy and the occurrence of placental abruption.

**Figure 12:**
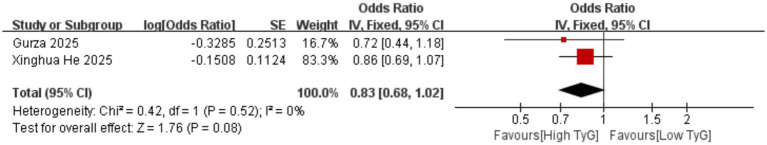
Forest plot showing the relationship between TyG index in early pregnancy and the occurrence of cesarean section.

**Figure 13:**
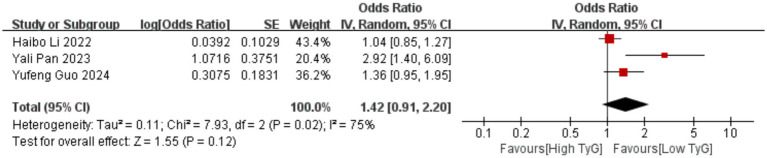
Forest plot showing the relationship between TyG index in early pregnancy and the occurrence of fetal distress.

1. Correlation between early pregnancy TyG index and PTB: Six studies evaluated the correlation between the TyG index (as a categorical variable) and PTB occurrence. No significant heterogeneity existed among the included results (*I^2^* = 39%), thus employing a fixed-effects model. Results indicated that compared with low TyG index in early pregnancy, high TyG index was associated with a higher risk of PTB, with a statistically significant difference [OR = 1.33, 95% CI (1.22, 1.44), *p* < 0.00001] See Figure 5.
2. Correlation between early pregnancy TyG index and LGA/SGA: Six studies evaluated the correlation between TyG index as a categorical variable and LGA occurrence. Significant heterogeneity existed among the included results (*I^2^* = 66%). Therefore, a random-effects model was used for analysis. Results showed that compared with low TyG index in early pregnancy, high TyG index was associated with a higher risk of LGA, with a statistically significant difference [OR = 1.70, 95% CI (1.37, 2.10), *p* < 0.00001]; Two studies evaluated the association between the TyG index as a categorical variable and SGA. No heterogeneity was observed among the included results (*I^2^* = 0%), thus a fixed-effects model was employed. Results indicated that the likelihood of SGA was similar between pregnant women with low and high TyG indices in early pregnancy, with no statistically significant difference [OR = 0.84, 95% CI (0.65, 1.10), *p* = 0.20] See Figures 6, 7.
3. Correlation between TyG index in early pregnancy and LBW: Five studies evaluated the correlation between TyG index as a categorical variable and LBW occurrence. Significant heterogeneity existed among the included results (*I^2^* = 83%). Therefore, a random-effects model was used for analysis. Results indicated no association between high TyG index in early pregnancy and LBW occurrence, with no statistically significant difference [OR = 1.30, 95% CI (0.84, 2.03), *p* = 0.24] See Figure 8.
4. Correlation between TyG index in early pregnancy and macrosomia: Four studies evaluated the correlation between the TyG index as a categorical variable and macrosomia occurrence. Significant heterogeneity existed among the included results (*I^2^* = 62%), necessitating a random-effects model analysis. Results indicated that compared with low TyG index in early pregnancy, high TyG index was associated with a statistically significant increased risk of macrosomia [OR = 1.41, 95% CI (1.02, 1.94), *p* = 0.04] See Figure 9.
5. Association between early pregnancy TyG index and PPROM, placental abruption, cesarean section, and fetal distress: Three studies evaluated the association between the TyG index as a categorical variable and PPROM. No significant heterogeneity existed among the included results (*I^2^* = 0%), thus a fixed-effect model was applied. Results indicated no association between high TyG index in early pregnancy and preterm premature rupture of membranes (OR = 1.08, 95% CI (0.94, 1.24), *p* = 0.30); Two studies evaluated the association between TyG index as a categorical variable and placental abruption. No significant heterogeneity was observed among the included results ^(^*I^2^* = 0%), thus a fixed-effect model was employed. The results indicated no statistically significant difference [OR = 1.25, 95% CI (0.80, 1.95), *p* = 0.32]. Two studies evaluated the TyG index as a categorical variable for its association with cesarean section occurrence. No significant heterogeneity existed among the included results (*I^2^* = 0%), thus a fixed-effect model was applied. The results indicated no statistically significant differences [OR = 0.83, 95% CI (0.68, 1.02), *p* = 0.08]. Three studies evaluated the association between TyG index as a categorical variable and fetal distress. Significant heterogeneity existed among the included results (*I^2^* = 75%), necessitating a random-effects model. Results showed no significant association between early pregnancy TyG index and fetal distress, with differences lacking statistical significance [OR = 1.17, 95% CI (0.99, 1.39), *p* = 0.07] See Figures 10–13.

#### 3.3.3 Subgroup analysis

Given the persistent high heterogeneity in some outcome measures, subgroup analysis was conducted to further investigate the sources of heterogeneity and determine the pooled effect sizes for each subgroup. Due to the limited number of studies using continuous variables as research parameters, corresponding subgroup analyses were not performed. Subgroup analyses were performed for outcomes where the TyG index served as a categorical variable. Studies were stratified by study type, geographic region, diagnostic criteria for pregnancy complications, and timing of TyG collection. Subgroup analyses were conducted separately for GDM, PE, GH, and APOs, with results presented in Table 2.

**Table 2:** Results of subgroup analysis.

Adjustment variables	Stratified subgroup	Number	P Heterogeneity	*I*^2^ Value (%)	Effect Model	Effect Size OR (95% CI)	*p*-value
GDM							
Study type	Retrospective cohort study	5	<0.00001	88	Random	2.45 [1.92, 3.13]	<0.00001
	Prospective cohort study	11	<0.00001	73	Random	2.59 [2.09, 3.20]	<0.00001
Country	Asia	14	<0.0001	70	Random	2.69 [2.36, 3.06]	<0.00001
	Latin America	2	0.06	72	Random	1.49 [0.84, 2.63]	0.17
GDM diagnostic criteria	IADPSG	12	<0.00001	84	Random	2.44 [2.10, 2.84]	<0.00001
	DIPSI	1	—	—	—	13.04[1.75,97.35]	0.01
	Guidelines for diagnosis and management of gestational Hyperglycemia (2022 Edition)	2	0.13	57	Random	2.79 [1.56, 4.99]	0.0006
	Carpenter and Coustan	1	—	—	—	5.62 [1.19, 26.44]	0.03
Indicator collection time	5–8	1	—	—	—	2.67 [1.17, 6.09]	0.02
	6–14	1	—	—	—	2.77 [2.52, 3.04]	<0.00001
	10–14	1	—	—	—	5.62 [1.19, 26.44]	0.03
	<12	2	0.63	0	Fixed	4.05 [2.08, 7.88]	<0.0001
	≤14	7	<0.00001	66	Random	2.13 [1.82, 2.50]	0.008
	<20	1	—	—	—	13.04 [1.75,97.35]	0.01
	9–14	1	—	—	—	3.16 [2.92, 3.42]	<0.00001
	6–10	1	—	—	—	2.44 [1.77, 3.36]	<0.00001
	6–12	1	—	—	—	3.59 [2.32, 5.57]	<0.00001
PE							
Study type	Retrospective cohort study	4	0.001	82	Random	1.88 [1.23, 2.86]	0.003
	Prospective cohort study	3	0.007	80	Random	1.32 [0.52, 3.31]	0.56
Country	Asia	6	<0.0001	81	Random	1.58 [1.06, 2.34]	0.02
	Latin America	1	—	—	—	2.38 [1.10, 5.15]	0.03
PE diagnostic criteria	ICD-10 O13	1	—	—	—	0.56 [0.30, 1.05]	0.07
	ACOG	5	0.002	77	Random	1.93 [1.32, 2.82]	0.0007
	ISSHP	1	—	—	—	1.49 [1.00, 2.22]	0.05
Indicator collection time	≤14	5	0.0005	80	Random	1.81 [0.97, 3.37]	0.06
	6–10	1	—	—	—	1.49 [1.00, 2.22]	0.05
	<20	1	—	—	—	1.31 [1.11, 1.55]	0.001
GH							
Study type	Retrospective cohort study	5	0.05	59	Random	1.97 [1.61, 2.40]	<0.00001
	Prospective cohort study	3	0.26	26	Fixed	1.39 [0.93, 2.09]	0.11
Country	Asia	7	0.01	63	Random	1.84 [1.50, 2.24]	<0.00001
	Latin America	1	—	—	—	1.66 [0.44, 6.26]	0.45
GH diagnostic criteria	ACOG	6	0.08	48	Fixed	1.95 [1.62, 2.35]	<0.00001
	ISSHP	1	—	—	—	1.87 [1.11, 3.15]	0.02
	ICD-10 O14	1	—	—	—	1.07 [0.69, 1.65]	0.76
Indicator collection time	≤14	5	0.24	19	Fixed	1.53 [1.24, 1.88]	<0.00001
	9–12	1	—	—	—	2.44 [1.71, 3.48]	<0.00001
	9–14	1	—	—	—	1.94 [1.72, 2.19]	<0.00001
	6–10	1	—	—	—	3.23 [1.72, 6.07]	0.0003
LGA							
Study type	Retrospective cohort study	2	0.11	60	Random	1.45 [1.22, 1.72]	<0.00001
	Prospective cohort study	4	0.15	44	Fixed	2.46 [1.53, 3.95]	0.0002
Country	Asia	5	0.007	72	Random	1.69 [1.35, 2.11]	<0.00001
	Latin America	1	—	—	—	2.10 [0.81, 5.44]	0.13
Indicator collection time	<12	2	0.44	0	Fixed	4.19 [2.07, 8.49]	<0.0001
	<13	1	—	—	—	1.86 [1.51, 2.29]	<0.00001
	≤14	2	0.33	0	Fixed	1.32 [1.08, 1.61]	0.007
	9–14	1	—	—	—	1.55 [1.41, 1.70]	<0.00001
Macrosomia							
Study type	Retrospective cohort study	3	0.21	36	Fixed	1.21 [0.97, 1.52]	0.09
	Prospective cohort study	1	—	—	—	1.97 [1.36, 2.86]	0.0004
Indicator collection time	≤14	2	0.42	0	Fixed	1.11 [0.86, 1.43]	0.41
	<8	1	—	—	—	1.97 [1.36, 2.86]	0.0004
	6–10	1	—	—	—	1.77 [1.05, 2.98]	0.03
PTB							
Study type	Retrospective cohort study	5	0.09	50	Fixed	1.33 [1.22, 1.44]	<0.00001
	Prospective cohort study	1	—	—	—	1.56 [0.75, 3.25]	0.23
Country	Asia	5	0.09	50	Fixed	1.33 [1.22, 1.44]	<0.00001
	Latin America	1	—	—	—	1.56 [0.75, 3.25]	0.23
Indicator collection time	≤14	3	0.96	0	Fixed	1.71 [1.36, 2.15]	<0.00001
	9–14	1	—	—	—	1.31 [1.17, 1.47]	<0.00001
	<20	1	—	—	—	1.18 [1.01, 1.38]	0.04
	6–10	1	—	—	—	1.73 [1.06, 2.82]	0.03
LBW							
Indicator collection time	≤14	2	0.5	0	Fixed	1.50 [1.15, 1.94]	0.002
	9–12	1	—	—	—	2.59 [1.25, 5.37]	0.01
	<20	1	—	—	—	0.84 [0.74, 0.95]	0.007
	6–10	1	—	—	—	0.59 [0.17, 2.05]	0.41
SGA							
Study type	Retrospective cohort study	1	—	—	—	0.86 [0.65, 1.14]	0.29
	Prospective cohort study	1	—	—	—	0.70 [0.31, 1.58]	0.39
Country	Asia	1	—	—	—	0.86 [0.65, 1.14]	0.29
	Latin America	1	—	—	—	0.70 [0.31, 1.58]	0.39
Fetal distress							
Study type	Retrospective cohort study	1	—	—	—	2.92 [1.40, 6.09]	0.004
	Prospective cohort study	2	0.2	39	Fixed	1.11 [0.93, 1.32]	0.25
Indicator collection time	≤14	2	0.2	39	Fixed	1.11 [0.93, 1.32]	0.25
	9–12	1	—	—	—	2.92 [1.40, 6.09]	0.004
PPROM							
Study Type	Retrospective cohort study	1	—	—	—	1.07 [0.83, 1.38]	0.6
	Prospective cohort study	2	0.24	29	Fixed	1.08 [0.91, 1.28]	0.37
Indicator collection time	≤14	2	0.24	29	Fixed	1.08 [0.91, 1.28]	0.37
	6–10	1	—	—	—	1.07 [0.83, 1.38]	0.6
Cesarean section							
Study type	Retrospective cohort study	1	—	—	—	0.86 [0.69, 1.07]	0.18
	Prospective cohort study	1	—	—	—	0.72 [0.44, 1.18]	0.19
Country	Asia	1	—	—	—	0.86 [0.69, 1.07]	0.18
	Latin America	1	—	—	—	0.72 [0.44, 1.18]	0.19
Indicator collection time	≤14	1	—	—	—	0.72 [0.44, 1.18]	0.19
	6–10	1	—	—	—	0.86 [0.69, 1.07]	0.18

Bolded values indicate *p* > 0.05.

### 3.4 Publication bias and sensitivity analysis

**Figure 14:**
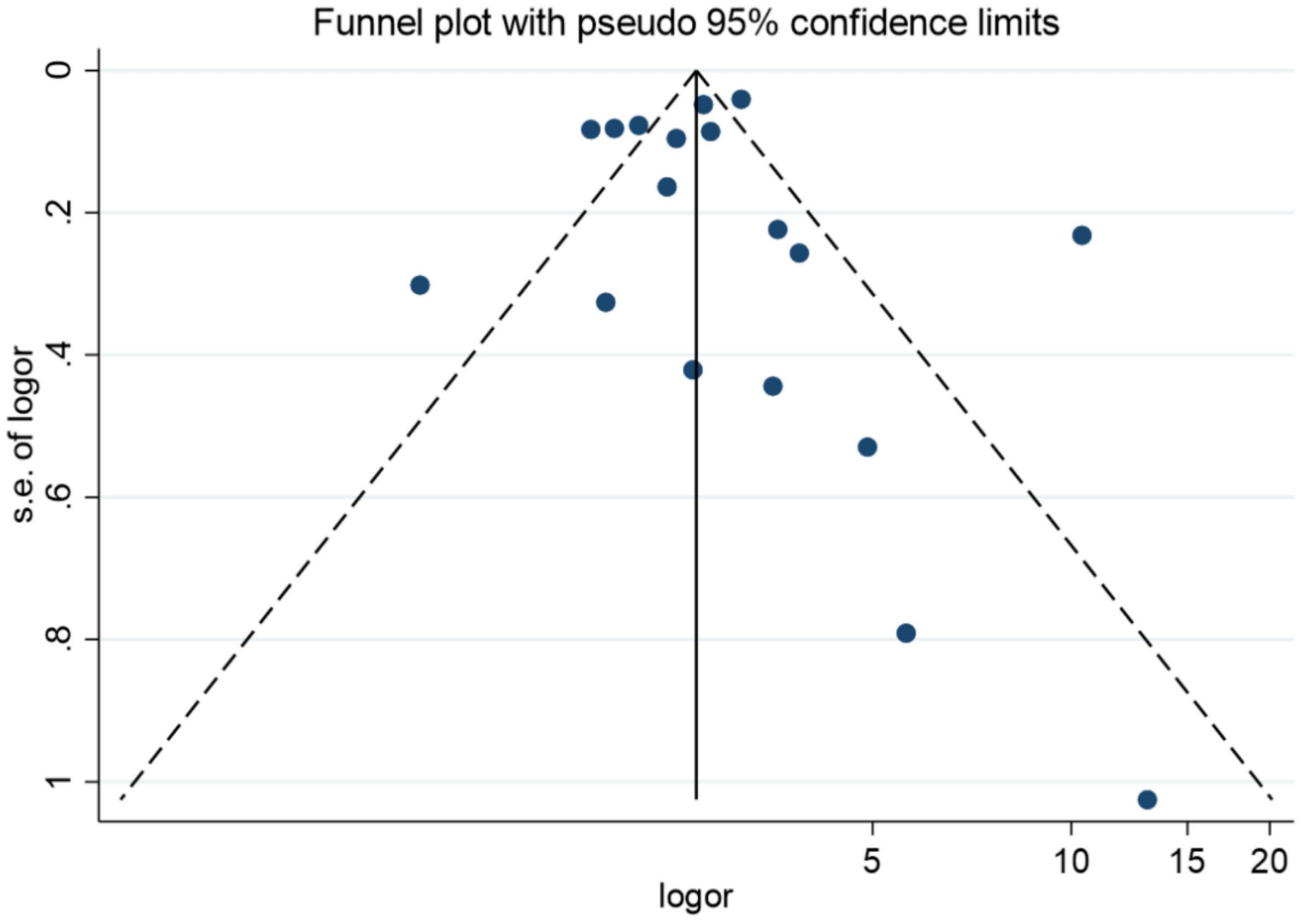
GDM publication bias funnel plot.

**Figure 15:**
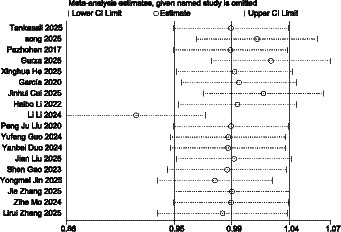
GDM sensitivity analysis.

For the 18 studies in this research that used GDM as the outcome indicator, a publication bias test was conducted. The funnel plot showed a generally symmetrical distribution, as shown in Figure 14. Further Egger’s test revealed *p* = 0.839 (*p* > 0.05), indicating that there was no significant publication bias in the meta-analysis, see Supplementary Figure S1. Sensitivity analysis was performed by sequentially excluding individual studies, Figure 15. After eliminating each study, the summary results did not show significant changes, except Li Li 2024, indicating that the results were basically stable, Li’s study possibly as a major source of heterogeneity in the research.

## 4 Discussion

### 4.1 Results analysis

This study, utilizing data from observational studies, corroborates that an elevated TyG index in early pregnancy is significantly correlated with an increased risk of gestational diabetes mellitus (GDM), gestational hypertension (GH), preeclampsia (PE), preterm birth (PTB), large for gestational age (LGA), and macrosomia. To address the substantial heterogeneity among the results, subgroup analyses were conducted based on various study types, regions, disease diagnostic criteria, and the timing of TyG index collection. The findings indicated that the significant association between a high TyG index in early pregnancy and adverse pregnancy outcomes, such as GDM, GH, and LGA, was predominantly observed in the Asian population. Although no statistical significance was detected in the Latin American population, this was primarily due to the inclusion of only two studies from this region, resulting in reduced statistical power. Furthermore, significant differences exist in TyG index levels, insulin sensitivity, and the strength of the association between the TyG index and disease occurrence among different ethnic and regional populations (3), leading to the non-universality of TyG index cutoff values across various regions and ethnicities (35). Consequently, the current evidence is insufficient to dismiss the predictive value of the TyG index for pregnancy complications and adverse pregnancy outcomes in Latin America and other non-Asian regions. Future large-scale, multi-center prospective cohort studies in non-Asian regions are urgently required to further elucidate the optimal cutoff values and predictive value of the TyG index in diverse ethnic and regional populations. The subgroup analysis of diagnostic criteria further validated the robustness of the predictive efficacy of the TyG index and identified some sources of heterogeneity. The study revealed that when employing internationally authoritative and unified guidelines for disease diagnosis, the predictive effect was more pronounced. This suggests that clinical practitioners should adopt unified and recognized clinical diagnostic gold standards whenever possible to effectively reduce heterogeneity among studies and provide higher-quality evidence-based medical evidence for clinical decision-making. Regarding the timing of testing, although insulin resistance and lipid metabolism changes are more pronounced in the second trimester and the predictive efficacy of the TyG index is relatively enhanced, early prediction (before 20 weeks of gestation) can identify high-risk pregnant women earlier and facilitate timely intervention, thereby reducing the incidence of adverse outcomes (36). However, the included studies in this research did not standardize the collection time of the TyG index in early pregnancy, which increased heterogeneity among studies and diminished the accuracy of its predictive efficacy for specific adverse outcomes. Future rigorous longitudinal cohort studies with standardized time points are urgently needed to dynamically depict the changes in the TyG index in early pregnancy and clarify its exact temporal relationship with various adverse pregnancy outcomes.

GDM and HDP are the two most common metabolic disorders in women during pregnancy, posing serious threats to maternal and infant health (37). In recent years, the prevalence of GDM has risen significantly, with approximately one in six women worldwide now experiencing hyperglycemia during pregnancy. GDM carries long-term health risks for both mothers and infants. Women with GDM face a tenfold increased risk of developing type 2 diabetes and are more susceptible to cerebrovascular diseases later in life compared to women without GDM. Additionally, children born to women with GDM are more prone to obesity and impaired glucose metabolism (38, 39). Currently, GDM is typically diagnosed between 24 and 28 weeks of gestation, leaving limited time for clinical management. Therefore, early assessment and prediction of GDM risk are particularly crucial. Although the pathophysiological mechanisms of GDM are complex, IR remains a key mechanism contributing to its development. Other metabolic factors involved include impaired pancreatic *β*-cell function, genetic predisposition, adipokines, and inflammatory cytokines (40, 41). This meta-analysis demonstrates a significant pos itive correlation between elevated TyG index in early pregnancy and GDM occurrence, consistent with the meta-analysis results by Song et al. (42), confirming the distinct predictive role of elevated TyG index in early pregnancy for GDM. HDP refers to a group of pregnancy-specific disorders characterized by hypertension, primarily including GH, PE, eclampsia, chronic hypertension during pregnancy, and chronic hypertension with PE. This study focuses on GH and PE. PE is an idiopathic hypertensive disorder of pregnancy and represents one manifestation of worsening GH. PE induces systemic arteriolar spasm and reduced perfusion to vital organs, leading to systemic functional impairment in mothers (43). This meta-analysis demonstrates that elevated TyG index in early pregnancy is significantly positively correlated with the occurrence of both GH and PE. Mechanistically, IR disrupts insulin signaling and contributes to chronic nutritional excess, collectively impairing vascular endothelial function. This leads to vasoconstriction, reduced nitric oxide (NO) and prostaglandin E2 (PGE2) synthesis, and heightened oxidative stress. Concurrently, IR induces systemic inflammatory responses and releases inflammatory adipokines, compounding oxidative endothelial damage (44), exacerbating maternal vascular dysfunction (45), ultimately leading to GH and PE (46). In summary, monitoring the TyG index during early pregnancy can predict the occurrence of maternal complications such as GDM, GH, and PE. Timely and effective clinical intervention can significantly reduce the risk of pregnancy complications and APOs.

APOs encompass PTB, PPROM, fetal distress, placental abruption, LGA/SGA, and macrosomia, affecting approximately 30% of pregnant women globally (47). Timely identification of metabolic disorders during pregnancy is crucial for reducing APOs incidence and even interrupting the intergenerational transmission of metabolic disorders (48). This study summarized the relationship between the TyG index in early pregnancy and the occurrence of APOs, revealing a significant positive correlation between a high TyG index in early pregnancy and the occurrence of macrosomia (birth weight > 4,000 g) and LGA (birth weight ≥ the 90th percentile for normal newborns of the same gestational age). Birth weight is generally considered a crucial indicator of fetal and neonatal health, and one of the modifiable factors affecting fetal growth in early pregnancy is IR. Research indicates that IR serves as a physiological adaptive mechanism to enhance fetal nutrient supply, while also constituting a necessary pathophysiological mechanism for the development of macrosomia and LGA (49). IR accelerates the maturation of the *β*-cell stimulation-secretion coupling mechanism, prompting the fetal pancreas to produce high levels of insulin. This, in turn, accelerates fetal growth and development. The combination of this high insulin and hyperglycemic state elevates amino acid and fatty acid levels in maternal blood, leading to excessive transfer of nutrients to the fetus via the placenta (50, 51). The accumulation of nutrients stimulates fetal pancreatic beta cells to secrete insulin, resulting in fetal hyperinsulinemia. This promotes excessive growth of insulin-sensitive tissues (such as the liver, adipose tissue, and heart), ultimately leading to macrosomia and LGA (52). PTB represents a major global public health challenge in perinatal medicine, directly impacting both short-term and long-term neonatal survival quality, with a worldwide incidence rate of 7.3% (53). This meta-analysis indicates that elevated TyG index in early pregnancy is also associated with increased maternal PTB risk. IR-induced maternal dysregulation of glucose and lipid metabolism promotes synthesis of plasminogen activator, reduces local fibrin degradation rates, and induces localized placental thrombosis. This leads to fetal hypoperfusion, impaired growth and development, and ultimately PTB occurrence (54). IR also excessively activates inflammatory responses, causing inflammatory mediators to damage placental vascular endothelial cells and impair their endothelial function. This leads to placental vasoconstriction and reduced blood perfusion, affecting fetal oxygen and nutrient uptake. Ultimately, this results in fetal growth restriction, fetal distress, or even PTB (55). This meta-analysis found no association between elevated TyG index in early pregnancy and fetal distress, placental abruption, PPROM, SGA, cesarean section, or LBW. However, studies indicate that IR exacerbates oxidative stress and lipid peroxidation, correlating with PPROM and the inflammatory cascade triggering labor (56). Furthermore, IR affects placental trophoblast invasion and vascular remodeling, leading to placental perfusion insufficiency and impaired nutrient transport (57). Theoretically, this may increase the likelihood of the aforementioned APOs. However, no association was found in this study, potentially due to the limited number of studies included for these outcome measures, which may introduce inaccuracies. Future research should expand the depth of investigation into these adverse pregnancy outcomes.

Current research consensus identifies the high-insulin euglycemic clamp as the gold standard for assessing IR. However, its clinical application is limited due to high costs and other drawbacks. In recent years, several simplified models for insulin sensitivity have been developed, such as Homeostasis Model Assessment of Insulin Resistance (HOMA-IR) and the TyG Index, which can be used to evaluate IR in epidemiological studies (58). Compared to the HOMA-IR model, the TyG index is more widely applied in clinical settings and can accurately predict IR and assess pregnancy-related metabolic disorders (59). Therefore, the TyG index can serve as an easily measurable, low-cost, and intervention-eligible alternative indicator for IR, enabling early identification of high-risk pregnant populations, assessment of pregnancy complication risks, and prediction of APOs.

### 4.2 Limitations of the study

While this study comprehensively evaluates the predictive value of the TyG index, the following limitations also exist: Although the selected models underwent thorough adjustment for confounding factors through multivariate analysis, residual confounding persists, such as intrauterine infection, cervical insufficiency, maternal diet, and exercise habits may influence final outcomes. Secondly, most studies included in this meta-analysis were conducted primarily on Asian populations, with only two studies involving non-Asian populations. Given the significant physiological differences in metabolic characteristics and insulin resistance patterns across regions and ethnic groups, triglyceride levels and the incidence of adverse pregnancy outcomes may vary. Therefore, the generalizability of the study conclusions to other regions and ethnic groups requires further validation. Additionally, the absence of standardized cutoff values for the TyG index internationally has hindered its uniform application in cross-regional and cross-ethnic screening for adverse pregnancy outcomes, thereby increasing the difficulty for clinicians to accurately predict adverse pregnancy outcomes. Finally, although the inclusion criteria defined the measurement time for the TyG index as before 20 weeks of gestation, the substantial temporal gaps in data collection across studies resulted in significant interstudy heterogeneity, which to some extent compromised the accuracy of the findings.

### 4.3 Research prospects

Large-scale, multicenter studies are urgently needed to validate the association between elevated TyG index and pregnancy complications/APOs across diverse populations, while optimizing the threshold setting for the TyG index to enhance its predictive efficacy. Longitudinal studies should track the dynamic changes of the TyG index during pregnancy to reveal its evolving relationship with metabolic and inflammatory responses. Future research may also integrate the TyG index with emerging biomarkers (dipokines, inflammatory factors) for combined prediction of pregnancy complications and APOs. Finally, it is recommended to incorporate the TyG index into routine prenatal screening. Healthcare providers can then implement nutritional counseling, medication, and enhanced monitoring for pregnant women at high risk of pregnancy complications, aiming to improve the identification rate of high-risk pregnancies and enhance maternal and infant outcomes.

## 5 Summary

In summary, the results of this study indicate that the TyG index in early pregnancy is significantly positively correlated with the occurrence of GDM, GH, PE, PTB, LGA, and macrosomia. As a simple and reliable surrogate marker, the TyG index can be used for the early diagnosis of pregnancy complications and APOs. In future clinical practice, monitoring the TyG index in early pregnancy can facilitate early identification and assessment of high-risk pregnancies, enabling timely targeted clinical interventions to reduce the incidence of maternal pregnancy complications and APOs.

## Data Availability

The original contributions presented in the study are included in the article/Supplementary material, further inquiries can be directed to the corresponding author.
