# Supplementary material for: The correlation between triglyceride-glucose index in early pregnancy (<20 weeks) and pregnancy complications and adverse pregnancy outcomes: a systematic review and meta-analysis
Source: Front Med (Lausanne). 2026 Apr 23;13:1811358. doi: 10.3389/fmed.2026.1811358 (PMC13149397; doi:10.3389/fmed.2026.1811358)
Supplement: Supplementary file 1 [file Table_1.docx]

| **TABLE S1** The Newcastle-Ottawa quality assessment scale of the included cohort studies. | | | | | | | | | | | | |
| --- | --- | --- | --- | --- | --- | --- | --- | --- | --- | --- | --- | --- |
| Study | Selection | | | |  | Comparability | |  | Assessment of outcome | | | Total score |
|  | Representativeness of exposure arm(s) | Selection of the comparative arm(s) | Origin of exposure source | Demonstration that outcome of interest was not present at start of study |  | Studies controlling the most important factors | Studies controlling the other main factors |  | Assessment of outcome with independency | Adequacy of follow-up length | Lost to follow-up acceptable |  |
| Tankasali 2025 | 1 | 1 | 1 | 1 |  | 1 | 0 |  | 0 | 1 | 1 | 7 |
| song 2025 | 1 | 1 | 1 | 1 |  | 1 | 0 |  | 1 | 1 | 1 | 8 |
| Pazhohan 2017 | 1 | 1 | 1 | 1 |  | 0 | 0 |  | 1 | 1 | 1 | 7 |
| Gurza 2025 | 1 | 1 | 1 | 1 |  | 0 | 0 |  | 1 | 1 | 1 | 7 |
| Huali Lin 2023 | 1 | 1 | 1 | 1 |  | 1 | 0 |  | 1 | 1 | 1 | 8 |
| Xinghua He 2025 | 1 | 1 | 1 | 1 |  | 1 | 0 |  | 1 | 1 | 1 | 8 |
| García 2020 | 1 | 1 | 1 | 1 |  | 1 | 0 |  | 1 | 1 | 1 | 8 |
| Jinhui Cui 2025 | 1 | 1 | 1 | 1 |  | 0 | 0 |  | 1 | 1 | 1 | 7 |
| Haibo Li 2022 | 1 | 1 | 1 | 1 |  | 1 | 0 |  | 1 | 1 | 1 | 8 |
| Yali Pan 2023 | 1 | 1 | 1 | 1 |  | 0 | 0 |  | 1 | 1 | 1 | 7 |
| Li Li 2024 | 1 | 1 | 1 | 1 |  | 0 | 0 |  | 1 | 1 | 1 | 7 |
| Peng 2020 | 1 | 1 | 1 | 1 |  | 1 | 0 |  | 1 | 1 | 1 | 8 |
| Guo 2024 | 1 | 1 | 1 | 1 |  | 1 | 0 |  | 1 | 1 | 0 | 7 |
| Duo Beiyan 2024 | 1 | 1 | 1 | 1 |  | 0 | 0 |  | 1 | 1 | 1 | 7 |
| Liu Jian 2025 | 1 | 1 | 1 | 1 |  | 1 | 0 |  | 1 | 1 | 0 | 7 |
| Jie Zhang 2025 | 1 | 1 | 1 | 1 |  | 1 | 0 |  | 1 | 1 | 0 | 7 |
| Zihe Mo 2024 | 1 | 1 | 1 | 1 |  | 1 | 0 |  | 1 | 1 | 1 | 8 |
| Zhang 2025 | 1 | 1 | 1 | 1 |  | 1 | 0 |  | 1 | 1 | 1 | 8 |
| Dan Zhao 2024 | 1 | 1 | 1 | 1 |  | 1 | 0 |  | 1 | 1 | 1 | 8 |
| Qiong Li 2025 | 1 | 1 | 1 | 1 |  | 1 | 0 |  | 1 | 1 | 1 | 8 |
| Gao C 2023 | 1 | 0 | 1 | 1 |  | 1 | 0 |  | 1 | 1 | 1 | 7 |
| Han Wenli 2025 | 1 | 1 | 1 | 1 |  | 0 | 0 |  | 1 | 1 | 1 | 7 |
| Jin Yongmei 2025 | 1 | 0 | 1 | 1 |  | 1 | 0 |  | 1 | 1 | 1 | 7 |
