# Supplementary material for: The correlation between triglyceride-glucose index in early pregnancy (<20 weeks) and pregnancy complications and adverse pregnancy outcomes: a systematic review and meta-analysis
Source: Front Med (Lausanne). 2026 Apr 23;13:1811358. doi: 10.3389/fmed.2026.1811358 (PMC13149397; doi:10.3389/fmed.2026.1811358)
Supplement: Supplementary file 3 [file Table_3.docx]

**PubMed 80**

#1 (((triglyceride glucose index[Title/Abstract]) OR (TyG index[Title/Abstract])) OR (TyG[Title/Abstract])) OR ((("Glucose"[Mesh]) OR (((((((((GLU[Title/Abstract]) OR (Glc[Title/Abstract]))OR (Amylaceum[Title/Abstract])) OR (dextrose[Title/Abstract])) OR(D-Glucose[Title/Abstract]))OR(Dextrose,Anhydrous[Title/Abstract]))OR(Glucose,(L)-Isomer[Title/Abstract]))OR(L-Glucose[Title/Abstract]))OR(Glucose Monohydrate[Title/Abstract])))AND(((((Triacylglycero[Title/Abstract])OR(Triglyceride*[Title/Abstract])) OR (TG[Title/Abstract])) OR (TAG[Title/Abstract])) OR ("Triglycerides"[Mesh])))

#2 (((((((((((((((((First Pregnancy Trimester[Title/Abstract]) OR (First Pregnancy Trimesters[Title/Abstract])) OR (Pregnancy Trimesters, First[Title/Abstract])) OR (Pregnancy, First Trimester[Title/Abstract])) OR (First Trimester Pregnancies[Title/Abstract])) OR (First Trimester Pregnancy[Title/Abstract])) OR (Pregnancies, First Trimester[Title/Abstract])) OR (Trimester, First[Title/Abstract])) OR (First Trimester[Title/Abstract])) OR (First Trimesters[Title/Abstract])) OR (Trimesters, First[Title/Abstract])) OR (Early Placental Phase[Title/Abstract])) OR (Early Placental Phases[Title/Abstract])) OR (Phase, Early Placental[Title/Abstract])) OR(Phases,Early Placental[Title/Abstract]))OR(Placental Phase, Early[Title/Abstract])) OR (Placental Phases, Early[Title/Abstract])) OR ("Pregnancy Trimester, First"[Mesh])

#3((((((((((((((((((((((Pregnancy-Induced Hypertension[Title/Abstract])OR (Gestational Hypertension[Title/Abstract]))OR(Hypertension, Gestational[Title/Abstract])) OR (Pregnancy Induced Hypertension[Title/Abstract])) OR (Hypertensions, Pregnancy Induced[Title/Abstract])) OR (Induced Hypertension, Pregnancy[Title/Abstract])) OR (Induced Hypertensions, Pregnancy[Title/Abstract])) OR (Transient Hypertension, Pregnancy[Title/Abstract]))OR(Hypertension, Pregnancy Transient[Title/Abstract]))OR(Pregnancy Transient Hypertension[Title/Abstract])) OR ("Hypertension, Pregnancy-Induced"[Mesh])) OR ((((((((Complication,Pregnancy[Title/Abstract])OR(Pregnancy Complication[Title/Abstract])) OR (Complications, Pregnancy[Title/Abstract])) OR (Adverse Birth Outcomes[Title/Abstract]))OR(Adverse Birth Outcome[Title/Abstract]))OR(Birth Outcome, Adverse[Title/Abstract])) OR (Outcome, Adverse Birth[Title/Abstract])) OR ("Pregnancy Complications"[Mesh]))) OR (((((((Diabetes Mellitus, Gestational[Title/Abstract]) OR (Gestational Diabetes Mellitus[Title/Abstract])) OR (Diabetes, Pregnancy-Induced[Title/Abstract])) OR (Diabetes, Pregnancy Induced[Title/Abstract])) OR (Pregnancy-Induced Diabetes[Title/Abstract])) OR (Gestational Diabetes[Title/Abstract])) OR ("Diabetes, Gestational"[Mesh]))) OR ((((((((((((((((((((((((((((((((Preeclampsia[Title/Abstract]) OR (Pregnancy Toxemias[Title/Abstract])) OR (Pregnancy Toxemia[Title/Abstract])) OR (Toxemia, Pregnancy[Title/Abstract])) OR (Edema-Proteinuria-Hypertension Gestosis[Title/Abstract])) OR (Edema Proteinuria Hypertension Gestosis[Title/Abstract])) OR (Gestosis, Edema-Proteinuria-Hypertension[Title/Abstract])) OR (Hypertension-Edema-Proteinuria Gestosis[Title/Abstract])) OR (Gestosis, Hypertension-Edema-Proteinuria[Title/Abstract])) OR (Hypertension Edema Proteinuria Gestosis[Title/Abstract])) OR (Proteinuria-Edema-Hypertension Gestosis[Title/Abstract])) OR (Gestosis, Proteinuria-Edema-Hypertension[Title/Abstract])) OR (Proteinuria Edema Hypertension Gestosis[Title/Abstract])) OR (EPH Complex[Title/Abstract])) OR (EPH Toxemias[Title/Abstract])) OR (EPH Toxemia[Title/Abstract])) OR (Toxemia, EPH[Title/Abstract])) OR (Toxemias, EPH[Title/Abstract])) OR (EPH Gestosis[Title/Abstract])) OR (Gestosis, EPH[Title/Abstract])) OR (Toxemias, Pregnancy[Title/Abstract])) OR (Toxemia Of Pregnancy[Title/Abstract])) OR (Of Pregnancies, Toxemia[Title/Abstract])) OR (Of Pregnancy, Toxemia[Title/Abstract])) OR (Pregnancies, Toxemia Of[Title/Abstract])) OR (Pregnancy, Toxemia Of[Title/Abstract])) OR (Toxemia Of Pregnancies[Title/Abstract])) OR (Preeclampsia Eclampsia 1[Title/Abstract])) OR (1s, Preeclampsia Eclampsia[Title/Abstract])) OR (Eclampsia 1s, Preeclampsia[Title/Abstract])) OR (Preeclampsia Eclampsia 1s[Title/Abstract])) OR ("Pre-Eclampsia"[Mesh]))) OR (((((((Births, Premature[Title/Abstract]) OR (Premature Births[Title/Abstract])) OR (Preterm Birth[Title/Abstract])) OR (Birth, Preterm[Title/Abstract])) OR (Births, Preterm[Title/Abstract])) OR (Preterm Births[Title/Abstract])) OR ("Premature Birth"[Mesh]))) OR (("Fetal Macrosomia"[Mesh]) OR (((Fetal Macrosomias[Title/Abstract]) OR (Macrosomias, Fetal[Title/Abstract])) OR (Macrosomia, Fetal[Title/Abstract])))) OR ((((((((((Low-Birth-Weight Infant[Title/Abstract]) OR (Infant, Low-Birth-Weight[Title/Abstract])) OR (Infants, Low-Birth-Weight[Title/Abstract])) OR (Low Birth Weight Infant[Title/Abstract])) OR (Low-Birth-Weight Infants[Title/Abstract])) OR (Low Birth Weight[Title/Abstract])) OR (Birth Weight, Low[Title/Abstract])) OR (Birth Weights, Low[Title/Abstract])) OR (Low Birth Weights[Title/Abstract])) OR ("Infant, Low Birth Weight"[Mesh]))) OR (((Nonreassuring Fetal Status[Title/Abstract]) OR (Fetal Status, Nonreassuring[Title/Abstract])) OR ("Fetal Distress"[Mesh]))) OR (((((((((((Fetal Membranes, Premature Rupture[Title/Abstract]) OR (Premature Rupture of Membrane (Pregnancy[Title/Abstract]))) OR (Membrane Premature Rupture (Pregnancy[Title/Abstract]))) OR (Membrane Premature Ruptures (Pregnancy[Title/Abstract]))) OR (PROM (Pregnancy[Title/Abstract]))) OR (Preterm Premature Rupture of Fetal Membranes[Title/Abstract])) OR (PPROM[Title/Abstract])) OR (Preterm Premature Rupture of the Membranes[Title/Abstract])) OR (Preterm PROM (Pregnancy[Title/Abstract]))) OR (PROM, Preterm (Pregnancy[Title/Abstract]))) OR ("Premature Rupture of Fetal Membranes"[Mesh]))) OR (((((Placental Abruption[Title/Abstract]) OR (Abruption, Placental[Title/Abstract])) OR (Abruptions, Placental[Title/Abstract])) OR (Placental Abruptions[Title/Abstract])) OR ("Abruptio Placentae"[Mesh]))) OR ("Infant, Small for Gestational Age"[Mesh])) OR (((((((((((((((High-Birth-Weight Infant[Title/Abstract]) OR (High-Birth-Weight Infants[Title/Abstract])) OR (Infant, High-Birth-Weight[Title/Abstract])) OR (Infants, High-Birth-Weight[Title/Abstract])) OR (Large-for-Gestational-Age Infant[Title/Abstract])) OR (Infant, Large-for-Gestational-Age[Title/Abstract])) OR (Infants, Large-for-Gestational-Age[Title/Abstract])) OR (Large-for-Gestational-Age Infants[Title/Abstract])) OR (Large for Gestational Age Infant[Title/Abstract])) OR (High Birth Weight Infant[Title/Abstract])) OR (LGA Infant[Title/Abstract])) OR (Infant, LGA[Title/Abstract])) OR (Infants, LGA[Title/Abstract])) OR (LGA Infants[Title/Abstract])) OR ("Infant, Large for Gestational Age"[Mesh]))) OR ((((((((((((Cesarean Sections[Title/Abstract]) OR (Abdominal Delivery[Title/Abstract])) OR (C-Section (OB[Title/Abstract]))) OR (C Section (OB[Title/Abstract]))) OR (C-Sections (OB[Title/Abstract]))) OR (Caesarean Section[Title/Abstract])) OR (Caesarean Sections[Title/Abstract])) OR (Delivery, Abdominal[Title/Abstract])) OR (Abdominal Deliveries[Title/Abstract])) OR (Deliveries, Abdominal[Title/Abstract])) OR (Postcesarean Section[Title/Abstract])) OR ("Cesarean Section"[Mesh]))

#4 #1 AND #2 AND #3

**Web of Science 68**

#1 triglyceride glucose index (Topic) OR TyG index (Topic) OR TyG (Topic)

#2 First Pregnancy Trimester (Topic) OR First Pregnancy Trimesters (Topic) OR Pregnancy Trimesters, First (Topic) OR Pregnancy, First Trimester (Topic) OR First Trimester Pregnancies (Topic) OR Pregnancies, First Trimester (Topic) OR Trimester*, First (Topic) OR Early Placental Phase* (Topic) OR First Trimester* (Topic) OR Phase*, Early Placental (Topic) OR Placental Phase*, Early (Topic) OR Pregnancy Trimester, First (Topic)

#3 First Pregnancy Trimester (Topic) OR First Pregnancy Trimesters (Topic) OR Pregnancy Trimesters, First (Topic) OR Pregnancy, First Trimester (Topic) OR First Trimester Pregnancies (Topic) OR Pregnancies, First Trimester (Topic) OR Trimester*, First (Topic) OR Early Placental Phase* (Topic) OR First Trimester* (Topic) OR Phase*, Early Placental (Topic) OR Placental Phase*, Early (Topic) OR Pregnancy Trimester, First (Topic) OR Pregnancy* Induced Hypertension (Topic) OR Gestational Hypertension (Topic) OR Hypertension, Gestational (Topic) OR Hypertensions, Pregnancy Induced (Topic) OR Induced Hypertension*, Pregnancy (Topic) OR Transient Hypertension, Pregnancy (Topic) OR Hypertension, Pregnancy Transient (Topic) OR Pregnancy Transient Hypertension (Topic) OR Hypertension, Pregnancy-Induced (Topic) OR Complication*,Pregnancy (Topic) OR Pregnancy Complication* (Topic) OR Adverse Birth Outcome* (Topic) OR Birth Outcome, Adverse (Topic) OR Outcome, Adverse Birth (Topic) OR Diabetes Mellitus, Gestational (Topic) OR Gestational Diabetes Mellitus (Topic) OR Diabetes, Pregnancy-Induced (Topic) OR Diabetes, Pregnancy Induced (Topic) OR Pregnancy-Induced Diabetes (Topic) OR Gestational Diabetes(Topic) OR Diabetes, Gestational (Topic) OR Preeclampsia (Topic) OR Pregnancy Toxemia* (Topic) OR Toxemia, Pregnancy (Topic) OR Edema-Proteinuria-Hypertension Gestosis (Topic) OR Edema Proteinuria Hypertension Gestosis (Topic) OR Gestosis, Edema-Proteinuria-Hypertension (Topic) OR Hypertension-Edema-Proteinuria Gestosis (Topic) OR Gestosis, Hypertension-Edema-Proteinuria (Topic) OR Hypertension Edema Proteinuria Gestosis (Topic) OR Proteinuria-Edema-Hypertension Gestosis (Topic) OR Gestosis, Proteinuria-Edema-Hypertension (Topic) OR Proteinuria Edema Hypertension Gestosis (Topic) OR EPH Complex (Topic) OR EPH Toxemia* (Topic) OR Toxemia*, EPH (Topic) OR EPH Gestosis (Topic) OR Gestosis, EPH (Topic) OR Toxemias, Pregnancy (Topic) OR Toxemia Of Pregnancy(Topic) OR Of Pregnancies, Toxemia (Topic) OR Of Pregnancy, Toxemia (Topic) OR Pregnancies, Toxemia Of (Topic) OR Pregnancy, Toxemia Of (Topic) OR Toxemia Of Pregnancies (Topic) OR Preeclampsia Eclampsia 1 (Topic) OR 1s, Preeclampsia Eclampsia (Topic) OR Eclampsia 1s, Preeclampsia (Topic) OR Preeclampsia Eclampsia 1s (Topic) OR Pre-Eclampsia (Topic) OR Births, Premature (Topic) OR Premature Births (Topic) OR PretermBirth (Topic) OR Birth*, Preterm (Topic) OR Preterm Births (Topic) OR Premature Birth (Topic) OR Fetal Macrosomia* (Topic) OR Macrosomia*, Fetal(Topic) OR Low-Birth-Weight Infant (Topic) OR Infant*, Low-Birth-Weight (Topic) OR Low Birth Weight Infant (Topic) OR Low-Birth-Weight Infants (Topic) OR Low Birth Weight (Topic) OR Birth Weight, Low (Topic) OR Birth Weights, Low (Topic) OR Low Birth Weights (Topic) OR infant, Low Birth Weight(Topic) OR Nonreassuring Fetal Status (Topic) OR Fetal Status, Nonreassuring(Topic) OR Fetal Distress (Topic) OR Fetal Membranes, Premature Rupture (Topic) OR Premature Rupture of Membrane (Pregnancy) (Topic) OR Membrane Premature Rupture (Pregnancy) (Topic) OR Membrane Premature Ruptures (Pregnancy) (Topic) OR PROM (Pregnancy) (Topic) OR Preterm Premature Rupture of Fetal Membranes (Topic) OR PPROM (Topic) OR Preterm Premature Rupture of the Membranes (Topic) OR Preterm PROM (Pregnancy) (Topic) ORPROM, Preterm (Pregnancy) (Topic) OR Premature Rupture of Fetal Membranes (Topic) OR Placental Abruption* (Topic) OR Abruption*, Placental (Topic) OR Abruptio Placentae (Topic) OR Infant, Small for Gestational Age(Topic) OR High-Birth-Weight Infant* (Topic) OR Infant*, High-Birth-Weight (Topic) OR Large-for-Gestational-Age Infant (Topic) OR Infant*, Large-for-Gestational-Age(Topic) OR Large-for-Gestational-Age Infants (Topic) OR Large for Gestational AgeInfant (Topic) OR High Birth Weight Infant (Topic) OR LGA Infant (Topic) OR Infant, LGA (Topic) OR Infants, LGA (Topic) OR LGA Infants (Topic) OR Infant, Large for Gestational Age (Topic) OR Cesarean Sections (Topic)ORAbdominal Delivery (Topic) OR C-Section* (OB) (Topic) OR C Section (OB) (Topic) OR Caesarean Section* (Topic) OR Delivery, Abdominal (Topic) OR Abdominal Deliveries (Topic) OR Deliveries, Abdominal (Topic) OR PostcesareanSection (Topic) OR Cesarean Section (Topic)

#4 #1 AND #2 AND #3

**Embase 43**

#1 'triglyceride glucose index'/exp OR 'tyg index':ti,ab,kw OR tyg:ti,ab,kw

#2 'first pregnancy trimester'/exp OR 'pregnancy, first trimester':ti,ab,kw OR 'pregnancy trimesters, first':ti,ab,kw OR 'first trimester*':ti,ab,kw OR 'trimester, first':ti,ab,kw OR 'first trimester pregnancies':ti,ab,kw OR 'placental phase*, early':ti,ab,kw OR 'early placental phase':ti,ab,kw

#3 'pregnancy-induced hypertension'/exp OR 'gestational hypertension':ti,ab,kw OR 'hypertension, gestational':ti,ab,kw OR 'pregnancy induced hypertension':ti,ab,kw OR 'hypertensions, pregnancy induced':ti,ab,kw OR 'induced hypertension, pregnancy':ti,ab,kw OR 'induced hypertensions, pregnancy':ti,ab,kw OR 'transient hypertension, pregnancy':ti,ab,kw OR 'hypertension, pregnancy transient':ti,ab,kw OR 'pregnancy transient hypertension':ti,ab,kw OR 'hypertension, pregnancy-induced':ti,ab,kw OR ‘complication,pregnancy’ OR 'pregnancy complication':ti,ab,kw OR 'complications, pregnancy':ti,ab,kw OR 'adverse birth outcomes':ti,ab,kw OR 'adverse birth outcome':ti,ab,kw OR 'birth outcome, adverse':ti,ab,kw OR 'outcome, adverse birth':ti,ab,kw OR 'pregnancy complications':ti,ab,kw OR 'diabetes mellitus, gestational' OR 'gestational diabetes mellitus':ti,ab,kw OR 'diabetes, pregnancy-induced':ti,ab,kw OR 'diabetes, pregnancy induced':ti,ab,kw OR 'pregnancy-induced diabetes':ti,ab,kw OR 'gestational diabetes':ti,ab,kw OR 'diabetes, gestational':ti,ab,kw OR 'preeclampsia'/exp OR 'pregnancy toxemias':ti,ab,kw OR 'pregnancy toxemia':ti,ab,kw OR 'toxemia, pregnancy':ti,ab,kw OR 'edema-proteinuria-hypertension gestosis':ti,ab,kw OR 'edema proteinuria hypertension gestosis':ti,ab,kw OR 'gestosis, edema-proteinuria-hypertension':ti,ab,kw OR 'hypertension-edema-proteinuria gestosis':ti,ab,kw OR 'gestosis, hypertension-edema-proteinuria':ti,ab,kw OR 'hypertension edema proteinuria gestosis':ti,ab,kw OR 'proteinuria-edema-hypertension gestosis':ti,ab,kw OR 'gestosis, proteinuria-edema-hypertension':ti,ab,kw OR 'proteinuria edema hypertension gestosis':ti,ab,kw OR 'eph complex':ti,ab,kw OR 'eph toxemias':ti,ab,kw OR 'eph toxemia':ti,ab,kw OR 'toxemia, eph':ti,ab,kw OR 'toxemias, eph':ti,ab,kw OR 'eph gestosis':ti,ab,kw OR 'gestosis, eph':ti,ab,kw OR 'preeclampsia'/exp OR 'pregnancy toxemias':ti,ab,kw OR 'pregnancy toxemia':ti,ab,kw OR 'toxemia, pregnancy':ti,ab,kw OR 'edema-proteinuria-hypertension gestosis':ti,ab,kw OR 'edema proteinuria hypertension gestosis':ti,ab,kw OR 'gestosis, edema-proteinuria-hypertension':ti,ab,kw OR 'hypertension-edema-proteinuria gestosis':ti,ab,kw OR 'gestosis, hypertension-edema-proteinuria':ti,ab,kw OR 'hypertension edema proteinuria gestosis':ti,ab,kw OR 'proteinuria-edema-hypertension gestosis':ti,ab,kw OR 'gestosis, proteinuria-edema-hypertension':ti,ab,kw OR 'proteinuria edema hypertension gestosis':ti,ab,kw OR 'eph complex':ti,ab,kw OR 'eph toxemias':ti,ab,kw OR 'eph toxemia':ti,ab,kw OR 'toxemia, eph':ti,ab,kw OR 'toxemias, eph':ti,ab,kw OR 'eph gestosis':ti,ab,kw OR 'gestosis, eph':ti,ab,kw OR 'births, premature' OR 'premature births':ti,ab,kw OR 'preterm birth':ti,ab,kw OR 'birth, preterm':ti,ab,kw OR 'births, preterm':ti,ab,kw OR 'preterm births':ti,ab,kw OR 'premature birth':ti,ab,kw OR 'fetal macrosomia'/exp OR 'fetal macrosomias':ti,ab,kw OR 'macrosomias, fetal':ti,ab,kw OR 'macrosomia, fetal':ti,ab,kw OR 'fetal macrosomia'/exp OR 'fetal macrosomias':ti,ab,kw OR 'macrosomias, fetal':ti,ab,kw OR 'macrosomia, fetal':ti,ab,kw OR 'low-birth-weight infant'/exp OR 'infant, low-birth-weight':ti,ab,kw OR 'infants, low-birth-weight':ti,ab,kw OR 'low birth weight infant':ti,ab,kw OR 'low-birth-weight infants':ti,ab,kw OR 'low birth weight':ti,ab,kw OR 'birth weight, low':ti,ab,kw OR 'birth weights, low':ti,ab,kw OR 'low birth weights':ti,ab,kw OR 'infant, low birth weight':ti,ab,kw OR 'nonreassuring fetal status'/exp OR 'fetal status, nonreassuring':ti,ab,kw OR 'fetal distress':ti,ab,kw OR 'fetal membranes, premature rupture'/exp OR 'premature rupture of membrane pregnancy':ti,ab,kw OR 'membrane premature rupture pregnancy':ti,ab,kw OR 'membrane premature ruptures pregnancy':ti,ab,kw OR 'prom pregnancy':ti,ab,kw OR 'preterm premature rupture of fetal membranes':ti,ab,kw OR pprom:ti,ab,kw OR 'preterm premature rupture of the membranes':ti,ab,kw OR 'preterm prom pregnancy':ti,ab,kw OR 'prom, preterm pregnancy':ti,ab,kw OR 'premature rupture of fetal membranes':ti,ab,kw OR 'placental abruption'/exp OR 'abruption, placental':ti,ab,kw OR 'abruptions, placental':ti,ab,kw OR 'placental abruptions':ti,ab,kw OR 'abruptio placentae':ti,ab,kw OR 'high-birth-weight infant' OR 'high-birth-weight infants':ti,ab,kw OR 'infant, high-birth-weight':ti,ab,kw OR 'infants, high-birth-weight':ti,ab,kw OR 'large-for-gestational-age infant':ti,ab,kw OR 'infant, large-for-gestational-age':ti,ab,kw OR 'infants, large-for-gestational-age':ti,ab,kw OR 'large-for-gestational-age infants':ti,ab,kw OR 'large for gestational age infant':ti,ab,kw OR 'high birth weight infant':ti,ab,kw OR 'lga infant':ti,ab,kw OR 'infant, lga':ti,ab,kw OR 'infants, lga':ti,ab,kw OR 'lga infants':ti,ab,kw OR 'infant, large for gestational age':ti,ab,kw OR 'infant, small for gestational age'/exp OR 'sga infant':ti,ab,kw OR 'infant, sga':ti,ab,kw OR 'infants, sga':ti,ab,kw OR 'sga infants':ti,ab,kw OR 'infant, small for gestational age':ti,ab,kw OR 'cesarean sections' OR 'abdominal delivery':ti,ab,kw OR ('c section':ti,ab,kw AND ob:ti,ab,kw) OR ('c sections':ti,ab,kw AND ob:ti,ab,kw) OR 'caesarean section':ti,ab,kw OR 'caesarean sections':ti,ab,kw OR 'delivery, abdominal':ti,ab,kw OR 'abdominal deliveries':ti,ab,kw OR 'deliveries, abdominal':ti,ab,kw OR 'postcesarean section':ti,ab,kw OR 'cesarean section':ti,ab,kw.

#4 #1 AND #2 AND #3

**The Cochrane Library 441**

#1 All Text=((triglyceride glucose index) OR (TyG index) OR (TyG))

#2 All Text=((First Pregnancy Trimester) OR (First Pregnancy Trimesters) OR (Pregnancy Trimesters, First) OR (Pregnancy, First Trimester) OR (First Trimester Pregnancies) OR (First Trimester Pregnancy) OR (Pregnancies, First Trimester)) OR (Trimester, First) OR (First Trimester) OR (First Trimesters) OR (Trimesters, First) OR (Early Placental Phase) OR (Early Placental Phases) OR (Phase, Early Placental) OR (Phases,Early Placental) OR (Placental Phase, Early) OR(Placental Phases, Early) OR (Pregnancy Trimester, First)

#3 All Text=((Pregnancy-Induced Hypertension) OR (Gestational Hypertension)OR(Hypertension, Gestational) OR (Pregnancy Induced Hypertension) OR (Hypertensions, Pregnancy Induced) OR (Induced Hypertension, Pregnancy) OR (Induced Hypertensions, Pregnancy) OR (Transient Hypertension, Pregnancy) OR (Hypertension, Pregnancy Transient) OR (Pregnancy Transient Hypertension)) OR (Hypertension, Pregnancy-Induced) OR (Complication,Pregnancy) OR (Pregnancy Complication) OR (Complications, Pregnancy)) OR (Adverse Birth Outcomes))OR(Adverse Birth Outcome))OR(Birth Outcome, Adverse)) OR (Outcome, Adverse Birth) OR (Pregnancy Complications) OR (Diabetes Mellitus, Gestational) OR (Gestational Diabetes Mellitus) OR (Diabetes, Pregnancy-Induced) OR (Diabetes, Pregnancy Induced) OR (Pregnancy-Induced Diabetes) OR (Gestational Diabetes) OR (Diabetes, Gestational) OR ((Preeclampsia) OR (Pregnancy Toxemias) OR (Pregnancy Toxemia) OR (Toxemia, Pregnancy) OR (Edema-Proteinuria-Hypertension Gestosis) OR (Edema Proteinuria Hypertension Gestosis) OR (Gestosis, Edema-Proteinuria-Hypertension) OR (Hypertension-Edema-Proteinuria Gestosis) OR (Gestosis, Hypertension-Edema-Proteinuria) OR (Hypertension Edema Proteinuria Gestosis) OR (Proteinuria-Edema-Hypertension Gestosis) OR (Gestosis, Proteinuria-Edema-Hypertension) OR (Proteinuria Edema Hypertension Gestosis) OR (EPH Complex) OR (EPH Toxemias) OR (EPH Toxemia) OR (Toxemia, EPH)) OR (Toxemias, EPH) OR (EPH Gestosis) OR (Gestosis, EPH) OR (Toxemias, Pregnancy) OR (Toxemia Of Pregnancy) OR (Of Pregnancies, Toxemia) OR (Of Pregnancy, Toxemia) OR (Pregnancies, Toxemia Of)) OR (Pregnancy, Toxemia Of) OR (Toxemia Of Pregnancies) OR (Preeclampsia Eclampsia 1) OR (1s, Preeclampsia Eclampsia) OR (Eclampsia 1s, Preeclampsia) OR (Preeclampsia Eclampsia 1s) OR (Pre-Eclampsia) OR ((Births, Premature) OR (Premature Births) OR (Preterm Birth) OR (Birth, Preterm) OR (Births, Preterm) OR (Preterm Births) OR (Premature Birth) OR (Fetal Macrosomia) OR (Fetal Macrosomias) OR (Macrosomias, Fetal) OR (Macrosomia, Fetal) OR (Low-Birth-Weight Infant) OR (Infant, Low-Birth-Weight) OR (Infants, Low-Birth-Weight) OR (Low Birth Weight Infant) OR (Low-Birth-Weight Infants) OR (Low Birth Weight) OR (Birth Weight, Low) OR (Birth Weights, Low) OR (Low Birth Weights) OR (Infant, Low Birth Weight) OR (Nonreassuring Fetal Status) OR (Fetal Status, Nonreassuring) OR (Fetal Distress) OR (Fetal Membranes, Premature Rupture) OR (Premature Rupture of Membrane (Pregnancy)) OR (Membrane Premature Rupture (Pregnancy)) OR (Membrane Premature Ruptures (Pregnancy)) OR (PROM (Pregnancy)) OR (Preterm Premature Rupture of Fetal Membranes) OR (PPROM)) OR (Preterm Premature Rupture of the Membranes) OR (Preterm PROM (Pregnancy)) OR (PROM, Preterm (Pregnancy)) OR (Premature Rupture of Fetal Membranes) OR (Placental Abruption) OR (Abruption, Placental) OR (Abruptions, Placental) OR (Placental Abruptions) OR (Abruptio Placentae) OR (Infant, Small for Gestational Age) OR (High-Birth-Weight Infant) OR (High-Birth-Weight Infants) OR (Infant, High-Birth-Weight) OR (Infants, High-Birth-Weight) OR (Large-for-Gestational-Age Infant) OR (Infant, Large-for-Gestational-Age)OR (Infants, Large-for-Gestational-Age) OR (Large-for-Gestational-Age Infants) OR (Large for Gestational Age Infant) OR (High Birth Weight Infant)) OR (LGA Infant) OR (Infant, LGA) OR (Infants, LGA) OR (LGA Infants) OR (Infant, Large for Gestational Age) OR (Cesarean Sections) OR (Abdominal Delivery) OR (C-Section (OB)) OR (C Section (OB)) OR (C-Sections (OB)) OR (Caesarean Section) OR (Caesarean Sections) OR (Delivery, Abdominal) OR (Abdominal Deliveries) OR (Deliveries, Abdominal) OR (Postcesarean Section) OR (Cesarean Section))

#4 #1 AND #2 AND #3
