# Supplementary figures and images for: The correlation between triglyceride-glucose index in early pregnancy (<20 weeks) and pregnancy complications and adverse pregnancy outcomes: a systematic review and meta-analysis
Source: Front Med (Lausanne). 2026 Apr 23;13:1811358. doi: 10.3389/fmed.2026.1811358 (PMC13149397; doi:10.3389/fmed.2026.1811358)

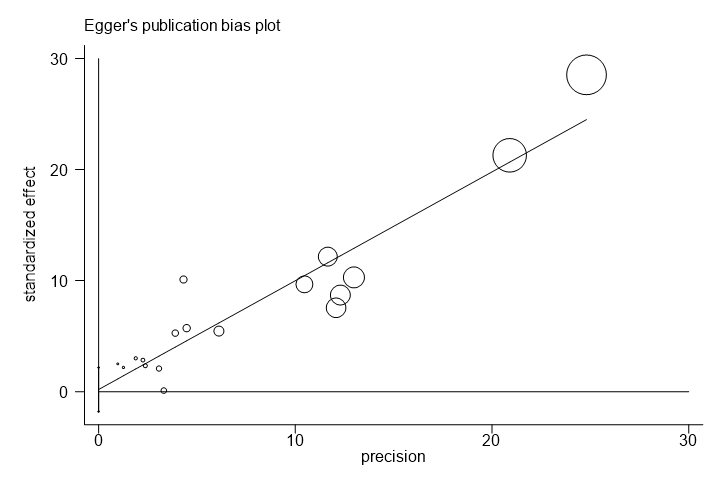

Supplement: Supplementary file 4 [file Image_1.tif]
